# Spatio-temporal patterns and associated factors of influenza-like illness outbreaks in Chinese mainland: a Bayesian modeling study

**DOI:** 10.1186/s12889-025-25615-7

**Published:** 2025-12-18

**Authors:** Xifei Guan, Zhixin Zhu, Feng Jiang, Yue Lei, Yancen Zhan, Xiuyang Li

**Affiliations:** 1 Department of Big Data in Health Science, Center for Clinical Big Data and Statistics, the Second Affiliated Hospital, College of Medicine, Zhejiang University, Hangzhou, Zhejiang 310058 China; 2 Department of Administrative Function, the Affiliated Hospital of Nantong University, Nantong, Jiangsu 226001 China

**Keywords:** Influenza-like illness outbreaks, Spatio-temporal analysis, Climate factors, Socioeconomic condition, Hierarchical bayesian model

## Abstract

**Background:**

China is one of the regions with high incidence of influenza, previous researches have primarily focused on the seasonal characteristics, spatio-temporal distribution, and associated influencing factors of influenza, while paying less attention to the public health significance of influenza-like illness (ILI) outbreaks. ILI is clinically defined as a syndrome characterized by fever accompanied by cough or sore throat. This case definition leads to distinct epidemiological characteristics, disease burden, and prevention strategies compared to laboratory-confirmed influenza. Currently, systematic epidemiological research on ILI outbreaks in Chinese mainland still has gaps. Therefore, a spatio-temporal modeling study was conducted to identify high-risk areas and potential risk factors for ILI outbreaks.

**Methods:**

The study utilized data on ILI outbreaks from the Chinese National Influenza Center. Spatial autocorrelation analysis, median center and standard deviational ellipse analysis were performed using ArcGIS 10.7 software to identify high-risk areas and spatial-temporal evolution of ILI outbreaks. Space-time scanning analysis was conducted using SaTScan 10.1.2 software to determine spatio-temporal clusters of ILI outbreaks. A Bayesian hierarchical model was adopted to explore the socioeconomic and meteorological factors influencing ILI outbreaks from a spatial-temporal perspective.

**Results:**

The outbreaks of ILI showed a distinct seasonality, with those in northern regions predominantly occurring during winter, whereas southern regions experienced more outbreaks, mainly in winter and spring. High clustering of ILI outbreaks was primarily concentrated in province levels such as Guangdong, Guangxi, Shandong, Jiangsu, Anhui, Zhejiang, and Fujian. The Bayesian model revealed that higher temperatures (RR = 0.958, 95% CI: 0.945–0.972), longer sunshine duration (RR = 0.871, 95% CI: 0.801–0.947), and higher wind speeds (RR = 0.820, 95% CI: 0.748–0.899) served as protective factors against ILI outbreaks, whereas surface pressure (RR = 1.005, 95% CI: 1.000-1.011) showed a positive correlation. Furthermore, regions with a higher proportion of males (RR = 1.022, 95% CI: 1.006–1.039), a greater proportion of population aged 14 and below (RR = 1.116, 95% CI: 1.054–1.179), higher GDP per capita (RR = 1.923, 95% CI: 1.212–3.047), and a larger floating population (RR = 1.943, 95% CI: 1.507–2.499) was also associated with a higher risk of ILI outbreaks.

**Conclusions:**

The study revealed distinct patterns and related influencing factors of ILI outbreaks in Chinese mainland from 2013 to 2022. Seasonality and spatial aggregation were its main characteristics. Temperature, sunshine duration, and wind speed were negatively correlated with the risk of ILI outbreaks, whereas surface pressure, the proportion of males, the proportion of population aged 14 and below, GDP per capita and floating population were positively correlated with the risk of ILI outbreaks. Relevant authorities should strengthen influenza surveillance in high-risk areas, optimize resource allocation, and enhance vaccination efforts to effectively prevent the exacerbation and spread of influenza outbreaks during peak seasons and in high-risk regions.

**Supplementary Information:**

The online version contains supplementary material available at 10.1186/s12889-025-25615-7.

## Introduction

Influenza is an acute respiratory disease caused by influenza viruses, classified as a Category C infectious disease. Influenza viruses can replicate extensively within the human body and are prone to mutations, which endows them with strong adaptability to various environments. Therefore, influenza typically presents with rapid onset, high contagiousness, universal susceptibility among the population, elevated local morbidity and may lead to the occurrence of pandemics [1]. Influenza can also cause severe respiratory symptoms and exacerbate symptoms of other chronic diseases. For instance, acute respiratory infections, including those caused by influenza viruses, can worsen asthma and chronic obstructive pulmonary disease (COPD), and acute influenza can also lead to congestive heart failure or decompensation in diabetic patients, increasing the risk of myocardial infarction and cerebrovascular accidents [2–5]. Influenza remains one of the world’s largest public health challenges. According to data from the World Health Organization (WHO), there are an estimated 1 billion cases of influenza globally each year, of which 3 to 5 million are severe cases, resulting in 290,000 to 650,000 influenza-related respiratory deaths [6], thereby imposing a substantial disease burden [7].

China is one of the regions with high incidence of influenza, where each outbreak of influenza can lead to elevated morbidity and mortality [8, 9]. Moreover, due to the diversity of factors such as population, economy, and environment across different regions in China, the prevention and control of influenza face significant challenges, concurrently exerting a formidable burden on China’s health system. In recent years, infectious respiratory diseases have become increasingly severe. China has experienced overlapping epidemics of multiple respiratory diseases, including Corona Virus Disease 2019 (COVID-19), influenza, mycoplasma pneumoniae infection, and others. Many individuals are suffering from recurrent infections and cross-infections, severely threatening human life and health. Studying the temporal and spatial evolution of influenza and related influencing factors can significantly alleviate the disease and economic burden in China.

Previous studies had shown that influenza in China exhibited a distinct seasonality [10], with a noticeable increase in the number of influenza infections during winter and spring [11]. The morbidity of influenza had been on a steady upward trend, displaying regional disparities [12, 13]. In tropical and subtropical regions, the morbidity of influenza exhibited multiple peaks throughout the year [14, 15]. Some researches had indicated that factors such as climate, latitude, and influenza vaccination coverage were associated with the seasonal pattern of influenza morbidity [12, 14]. However, few studies combine time series analysis with spatial heterogeneity to further explore the spatio-temporal evolution patterns and associated influencing factors of influenza outbreaks in China.

China established the National Influenza Surveillance Center in 1957, with sentinel hospitals effectively covering the whole country, thereby forming a relatively comprehensive influenza surveillance network. This study utilizes the surveillance data from influenza weekly reports issued by the Chinese National Influenza Center to conduct statistical analyses on influenza-like illness (ILI) cases and outbreak data across provinces (municipalities and autonomous regions) in Chinese mainland from 2013 to 2022. The aim is to elucidate the epidemiological characteristics, spatio-temporal evolution patterns, and related influencing factors of ILI outbreaks in China over the past decade, providing a theoretical basis to further refine the prevention and control of influenza in China.

## Methods

### Data collection

Data on the proportion of ILI cases, virological test results and ILI outbreaks from each province (municipality and autonomous region) in Chinese mainland were collected from the influenza weekly reports issued by the Chinese National Influenza Center (https://ivdc.chinacdc.cn/cnic/zyzx/lgzb/), spanning from Week 1 of 2013 to Week 52 of 2022.

The social and economic indicators utilized in the study were collected from the Chinese National Bureau of Statistics (https://www.stats.gov.cn/), encompassing total population, population density, gender ratio, proportion of population aged 14 and below, aging rate, urbanization rate, gross domestic product (GDP) per capita, disposable income per capita, number of health technicians, and floating population. The floating population refers to individuals who have been away from their registered residence for more than six months.

Meteorological data used in the research were obtained from the European Center for Medium-Range Weather Forecasts, which included temperature, surface pressure, precipitation, wind speed, relative humidity, and sunshine duration [16].

The map of China was sourced from the National Catalogue Service For Geographic Information (https://www.webmap.cn/main.do?method=index).

### Surveillance content and indicators

The National Influenza Surveillance Center in China comprises two components: the surveillance of ILI and ILI outbreaks. The surveillance work is conducted at sentinel hospitals across the country, where daily records are kept by department for both ILI and the total number of outpatient and emergency visits, with the data subsequently entered into the “China Influenza Surveillance Information System”. The ILI is defined as a patient who presents with fever (underarm temperature ≥ 38℃), accompanied by cough or sore throat, and lacks laboratory confirmation for any specific disease [17]. The ILI percentage (ILI%) refers to the proportion of ILI among all outpatients seen at a given healthcare facility over a specified period, expressed as a percentage of the total number of outpatient visits during the same period. ILI outbreak surveillance is carried out according to the “Guidelines for the Management of Outbreaks of ILI (2012 Edition)” [17]. An ILI outbreak is defined as an event where 10 or more ILI cases within the same region or facility over the course of one week, which is verified and confirmed by the county or district-level disease prevention and control institutions, and subsequently reported through the “China Influenza Surveillance Information System”. For outbreak surveillance, nasopharyngeal swab samples are collected from ILI patients who have not taken antiviral medications within three days of symptom onset. These samples are then subjected to etiological testing for influenza viruses through the established influenza surveillance network laboratories. The tests aim to identify the viral subtypes involved and perform viral isolation on positive specimens.

According to the National Technical Guidelines for Influenza Surveillance (2017 Edition), China divides regions conducting ILI surveillance into southern and northern areas. The southern region encompasses Shanghai, Jiangsu, Zhejiang, Anhui, Fujian, Jiangxi, Hubei, Hunan, Guangdong, Guangxi, Hainan, Chongqing, Sichuan, Guizhou and Yunnan. The northern region includes Beijing, Tianjin, Hebei, Shanxi, Inner Mongolia, Liaoning, Jilin, Heilongjiang, Shandong, Henan, Tibet, Shaanxi, Gansu, Qinghai, Ningxia, Xinjiang and Xinjiang Production and Construction Corps [18].

### Descriptive analysis

The data of ILI outbreaks, ILI% and etiological detection results in the north and south of Chinese mainland were sorted out, and the time series diagrams and percentage stacked bar charts were drawn to illustrate the long-term trend and the proportion of influenza subtypes. Concurrently, data on ILI outbreaks for each province (municipality and autonomous region) across the mainland were organized, and both a heat map and statistical map were drawn to depict the outbreak trends and regional distribution characteristics of ILI.

### Spatio-temporal analysis

#### Spatial autocorrelation analysis

Both global and local spatial autocorrelation analysis were applied to investigate the spatial patterns of ILI outbreaks in Chinese mainland during the period from 2013 to 2022. Global spatial autocorrelation was assessed by calculating the global Moran’s I Index, which provided an overall measure of spatial clustering or dispersion across the entire study region. The global Moran’s I Index ranged from − 1 to 1. A positive value of Moran’s I Index implied the presence of spatial autocorrelation, indicating that areas with similar ILI outbreak intensities tended to cluster together. This clustering could be characterized as a “high-high” or “low-low” pattern of spatial clustering. A negative value denoted negative spatial autocorrelation, indicating an alternating distribution pattern between adjacent elements with a “high-low” or “low-high” arrangement. A zero value suggested a lack of significant spatial autocorrelation, implying that ILI outbreak occurrences were more likely to be randomly distributed across the study area. In addition to the global assessment, local spatial autocorrelation was analyzed through the use of local indicators of spatial association (LISA) and the construction of a LISA cluster map, pinpointing the geographical distribution of ILI outbreak clusters. These clusters were classified into four types: high-high, high-low, low-low, and low-high, each representing different spatial configurations of ILI outbreak within the study areas [19].

#### Space-time scanning analysis

A retrospective scanning statistic was used to scan the spatio-temporal regions of ILI outbreaks in Chinese mainland from 2013 to 2022, identifying spatio-temporal clusters of ILI outbreaks. The maximum radius threshold for spatial windows was set at 15% of the population at risk, while the temporal window size for time clusters ranged from a single day to 50% of the study period. During the process of moving window scanning, the Log Likelihood Ratio (LLR) was computed for each selected window region based on its observed and expected values, enabling the determination of high-risk clusters. The relative risk (RR) was used to express the estimated risk ratio within and outside these clusters, with a high RR value indicating that individuals residing within a cluster faced a higher risk of infection compared to those living outside the cluster.

#### Mean center, median center, and standard deviational ellipse

The mean center is a valuable metric in spatial analysis in that it reveals the average x and y coordinates of all elements within the study region, serving to describe the concentrated location of these elements. However, the mean center is significantly influenced by extreme values, whereas the median center is a more robust measure of central tendency that is less sensitive to outliers. When the mean center returns a point at the mean x and y coordinates of the centroid of all elements, the median center uses an iterative algorithm to locate the point that minimizes the Euclidean distance among all elements within the study area. Given the substantial disparities in the distribution of ILI outbreaks across regions in Chinese mainland, the median center was utilized in this study. Standard deviational ellipses determine the principal axis orientation by calculating the standard deviations of x and y coordinates from the mean center, thereby revealing the elongation pattern and directional characteristics of element distribution.

The mean center was calculated using the following formula:

$$\overline{x} = \frac{\sum_{i=1}^{n} x_i}{n}, \quad \overline{y} = \frac{\sum_{i=1}^{n} y_i}{n}$$

The median center was calculated using the following formula:

$$y_{i+1}\;=\;(\sum_{j=1}^m\frac{x_j}{\left\|x_j-y_i\right\|})\;/(\sum_{j=1}^m\frac1{\left\|x_j-y_i\right\|})$$

The standard deviational ellipse was calculated using the following formula:

$$SDE_x=\sqrt{\frac{\sum_{i=1}^n{(x_1-\overline x)}^2}n},\;SDE_y=\sqrt{\frac{\sum_{i=1}^n{(y_1-\overline y)}^2}n}$$

Where *x*_*i*_ and *y*_*i*_ are the coordinates of element *i*, and *n* is equal to the total number of elements. *y*_*i*_ represents the current approximate median center point, while *y*_*i+1*_ denotes the next point. The first *y* point chooses the mean center, *x*_*j*_ signifies each individual point in the set, and ||*x*_*j*_ *- y*_*i*_ || refers to the Euclidean distance between *x*_*j*_ and *y*_*i*_. *SDE*_*x*_ and *SDE*_*y*_ represent coordinates of the center of the ellipse.

### Bayesian hierarchical spatio-temporal model

A Bayesian hierarchical spatial-temporal model was constructed based on the number of ILI outbreaks. It incorporated socioeconomic variables (encompassing population density, gender ratio, proportion of population aged 14 and below, aging rate, urbanization rate, GDP per capita, disposable income per capita, number of health technicians, and floating population) and meteorological variables (including temperature, surface pressure, precipitation, wind speed, relative humidity, and sunshine duration) as covariates to explore the related factors of ILI outbreaks. The variance inflation factor (VIF) was used to assess the multicollinearity among all covariates, with variables having VIF values less than 10 being selected for inclusion in the model [20]. In the study, the data on ILI outbreaks were overdispersed, with variances exceeding means, failing to meet the requirements of Poisson distribution, while the negative binomial regression model could account for such overdispersion in the data structure [21]. Additionally, due to a substantial number of data cells containing zero outbreak case counts, it was assumed that the number of ILI outbreaks followed the Zero-inflated Negative Binomial (ZINB) distributions:

$$P(Y_{it}\;=\;y_{it})=\left\{\begin{array}{l}p_{it}+(1-p_{it})\left(\frac{\alpha^{-1}}{\alpha^{-1}+\mu_{it}}\right)^{\alpha^{-1}},\;y_{it}=0\\(1-p_{it})\frac{\Gamma(y_{it}+\alpha^{-1})}{y_{it}\;!\Gamma(\alpha^{-1})}\left(\frac{\alpha^{-1}}{\alpha^{-1}+\mu_{it}}\right)^{\alpha^{-1}}\left(\frac{\mu_{it}}{\alpha^{-1}+\mu_{it}}\right)^{y_{it}},\;y_{it}>0\end{array}\right.$$

where *y*_*it*_ represents the number of observed ILI outbreaks in region *i* (*i* = 1,2,……,31) during time period *t* (*t* = Week 1 of 2013, Week 2 of 2013,……, Week 52 of 2022), *p*_*it*_ denotes the probability of zero occurrences in region *i* at time *t*, while *µ*_*i*_ is a parameter of the negative binomial distribution, signifying the expected number of ILI outbreaks in region *i* at time *t*, and *α* ^−1^ is the dispersion parameter.

Under the aforementioned assumptions, the logarithmic transformation of *µ*_*it*_ can be modeled as:

$$\begin{array}{c}Y_{it}\sim NB(\mu_{it})\\\log(\mu_{it})=\log(E_{it})+\theta_{it}\\\theta_{it}=\alpha_0+{\textstyle\sum_k^m}\beta_k\chi_k+\mu_i+\nu_i+\varphi_t+\psi_{it}\end{array}$$

where *E*_*it*_ denotes the number of expected ILI outbreaks, *θ*_*it*_ represents the log relative risk of ILI outbreak, *X*_*k*_ is the k-th variable in the model, *ɑ*_*0*_ is the intercept fixed effect; *β*_*k*_ denotes the fixed effect for the study variables, *u*_*i*_ represents the spatially structured random effect with mean zero and variance $\sigma^2_\mu$ , *v*_*i*_ is the spatially unstructured random effect with mean zero and variance$\sigma^2_\nu$ ,$\varphi_t$ denotes the temporal trend effect, and $\psi_{it}$ signifies the spatiotemporal interaction effect.

To estimate the posterior distribution under the Bayesian framework, the study assigned different prior distributions to the parameters. The Besag-York Mollie model was employed to model the spatial components *u*_*i*_ and *v*_*i*_. Specifically, the spatially structured random effect, *u*_*i*_, was modeled using the conditional autoregressive prior, while the spatially unstructured random effect, *v*_*i*_, was modeled assuming an independent and identically distributed normal distribution. $\varphi_t$ was modeled using a first-order random walk model. $\psi_{it}$ was utilized to account for any residual spatiotemporal variation not captured by the spatial or temporal main effects, and it was assumed to be independently and identically distributed as well. After establishing the framework of these models, the study incorporated covariates and employed the nested Laplace approximation algorithm within the R-INLA package of the R programming language (version 4.3.3) to estimate the parameters. In the research, three basic models were established first. Model I included socioeconomic and climate variables as well as spatial terms (*u*_*i*_ + *v*_*i*_). Model II added a temporal term ($\varphi_t$). Model III further incorporated the spatio-temporal interaction term ($\psi_{it}$ ). The deviance information criterion (DIC) was used to evaluate and compare the model fitness, and the best-performing model was selected. After identifying the optimal model, the study conducted both univariate and multivariate analyses of the covariates to comprehensively explore the influencing factors of ILI outbreaks.

### Statistical analysis methods

In the study, Excel 2021 software was used to organize the data and draw time series diagrams, percentage stacked bar charts and heat maps. ArcGIS 10.7 software was utilized to create statistical maps of ILI outbreaks and to perform spatial autocorrelation analysis, median center analysis, and standard deviational ellipse analysis. Space-time scanning analysis was performed using SaTScan 10.1.2 software to identify spatio-temporal clusters of ILI outbreaks. The R programming language version 4.3.3 was applied to establish the Bayesian spatio-temporal model to investigate the relevant factors of ILI outbreaks. The 95% Confidence intervals (CI) were utilized to represent the 2.5th to 97.5th percentiles of the posterior distribution for the ILI outbreaks. All analyses were two-sided tests and were considered statistically significant at *P* ≤ 0.05.

## Results

### Descriptive results

**Fig. 1:**
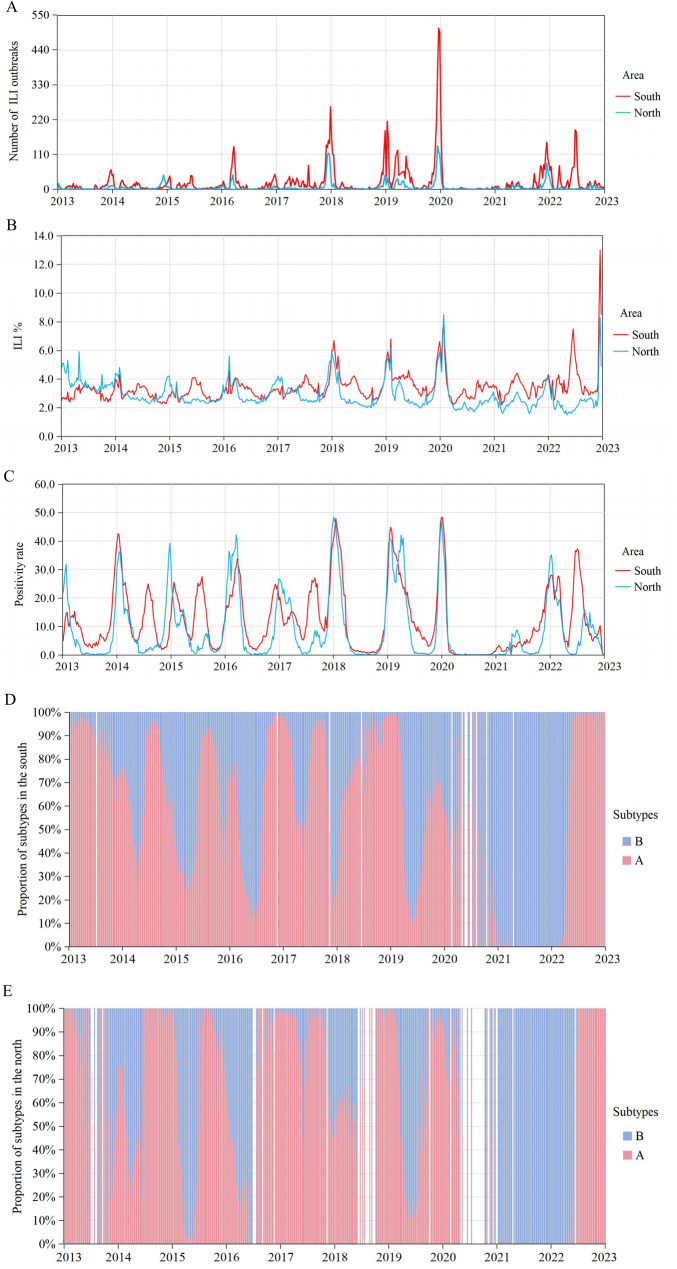
Epidemiological features of ILI outbreaks, ILI% and positive detection rate in Chinese mainland, 2013–2022. **A** The time series diagram for the number of ILI outbreaks in southern and northern. **B** The time series diagram for ILI% in southern and northern. **C** The time series diagram for laboratory positive detection rate in southern and northern. **D** The distribution of influenza subtypes in southern. **E** The distribution of influenza subtypes in northern

The time series diagrams for ILI% and laboratory positive detection rates in southern and northern regions of Chinese mainland from January 2013 to December 2022 were shown in Fig. 1, revealing distinct cyclical and seasonal patterns. Influenza was prevalent in the winter and spring in the North, while in the South, it tended to concentrate in the winter and summer seasons. Percentage stacked bar charts depicting the distribution of influenza subtypes in the southern and northern regions are presented in Fig. 1, as well. In the southern region, influenza A constituted a larger proportion, with influenza B exhibiting a slight advantage during the winter and spring seasons. In the northern region, influenza A similarly dominated, but the advantage of influenza B during spring was more pronounced. During 2021 to the spring of 2022, almost exclusively influenza B cases were observed in both the southern and northern regions.

**Fig. 2:**
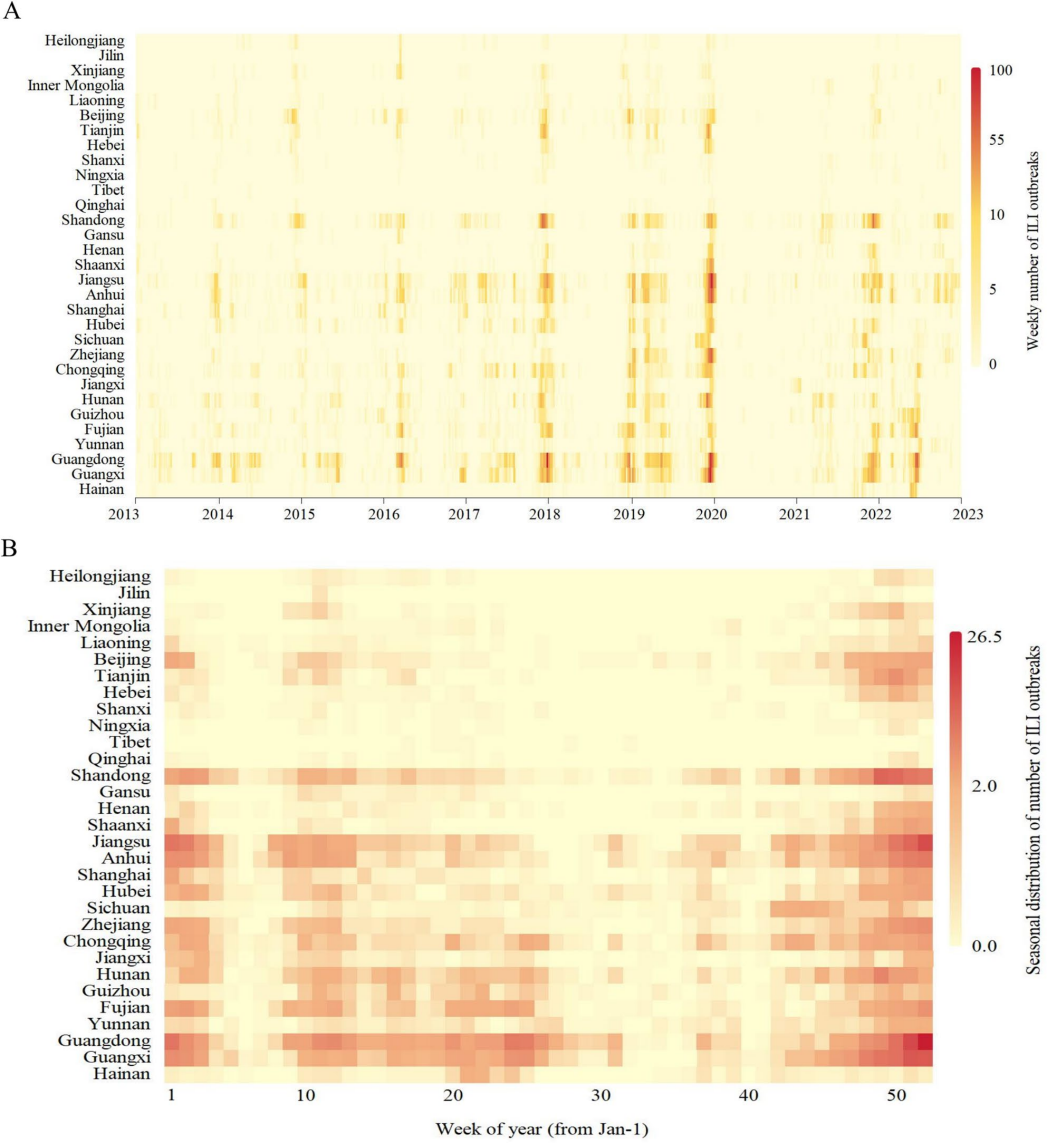
Heatmaps of ILI outbreaks data by Chinese province, 2013–2022. **A** The time series of weekly ILI outbreaks. **B** Average week distribution of ILI outbreaks, week 1 is the first week of January of each year

**Fig. 3:**
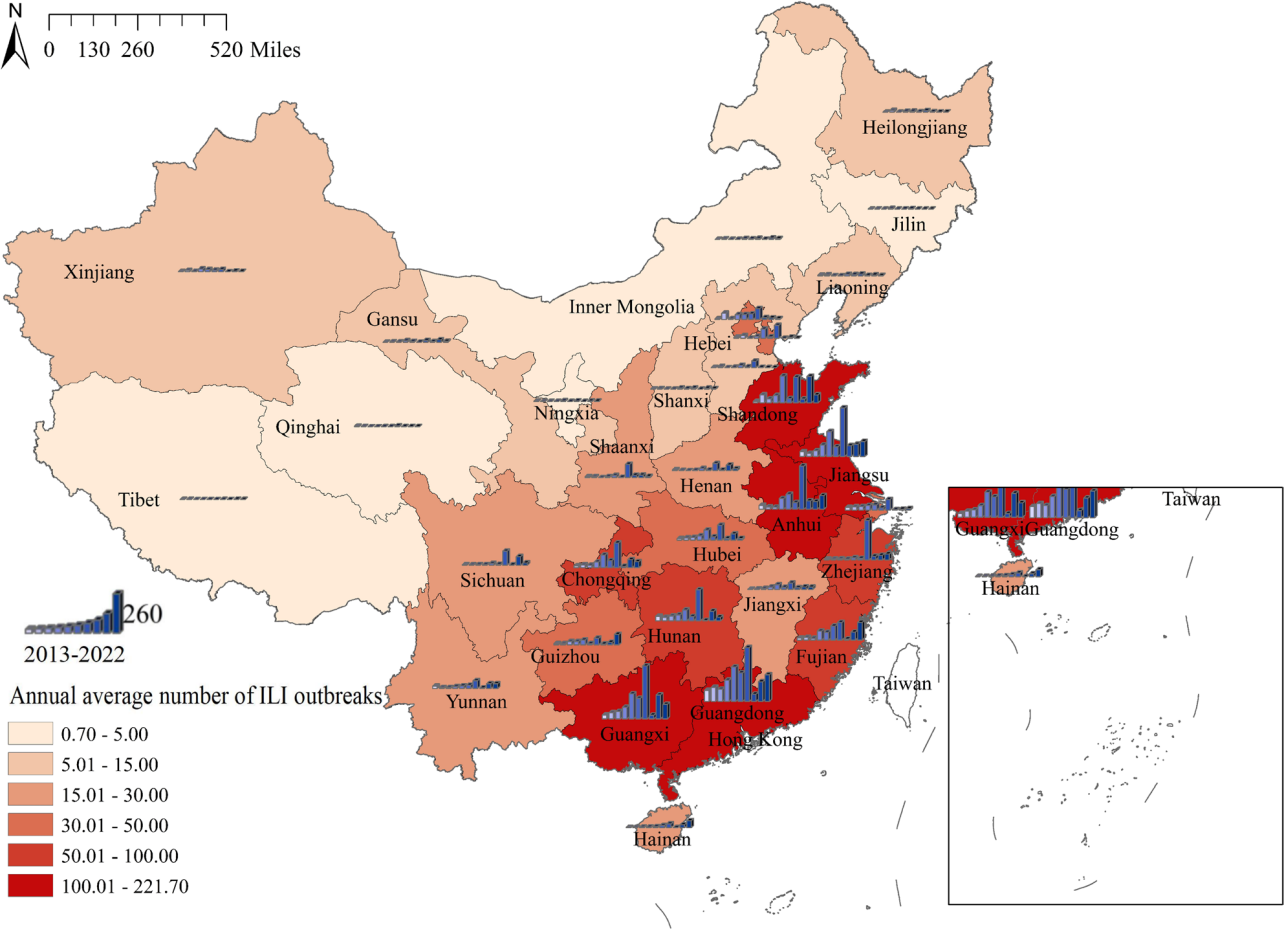
The spatial and temporal distribution of ILI outbreaks in Chinese mainland, 2013–2022

Time-series heat maps depicting ILI outbreaks across each province (municipality and autonomous region) in Chinese mainland were presented in Fig. 2. ILI outbreaks exhibited a distinct seasonality, with fewer occurrences in northern regions primarily concentrated during winter. In contrast, southern regions experienced a greater number of ILI outbreaks, predominantly in winter and spring. The spatial and temporal distribution of ILI outbreaks was illustrated in Fig. 3. The number of ILI outbreaks could be seen to show a fluctuating upward trend, reaching a maximum in 2019 and decreasing thereafter, which may be related to the outbreak of COVID-19. Notably, Guangdong, Guangxi, Shandong, Jiangsu, and Anhui reported significantly higher numbers of outbreaks compared to other areas.

### Spatio-temporal cluster

The spatial autocorrelation analysis was conducted on ILI outbreaks in Chinese mainland from 2013 to 2022. The results, as shown in Table 1, revealed that the global Moran’s I Index ranged from 0.19 to 0.43, with all *P* values < 0.05. This indicated a statistically significant positive spatial autocorrelation among ILI outbreaks across regions, suggesting a clustering pattern where regions with high or low outbreak frequencies tend to be spatially close to other regions with similar outbreak characteristics.

**Table 1:** Results of global Spatial autocorrelation analysis of ILI outbreaks in Chinese mainland, 2013–2022

Year	Moran’s I	Expected Index	Variance	z-score	*P*-value
2013	0.304	−0.033	0.009	3.499	0.001
2014	0.185	−0.033	0.010	2.169	0.030
2015	0.434	−0.033	0.011	4.456	0.000
2016	0.368	−0.033	0.012	3.716	0.000
2017	0.341	−0.033	0.013	3.321	0.001
2018	0.354	−0.033	0.010	3.781	0.000
2019	0.424	−0.033	0.013	3.942	0.000
2020	0.295	−0.033	0.012	3.061	0.002
2021	0.296	−0.033	0.012	2.980	0.003
2022	0.417	−0.033	0.012	4.051	0.000

**Fig. 4:**
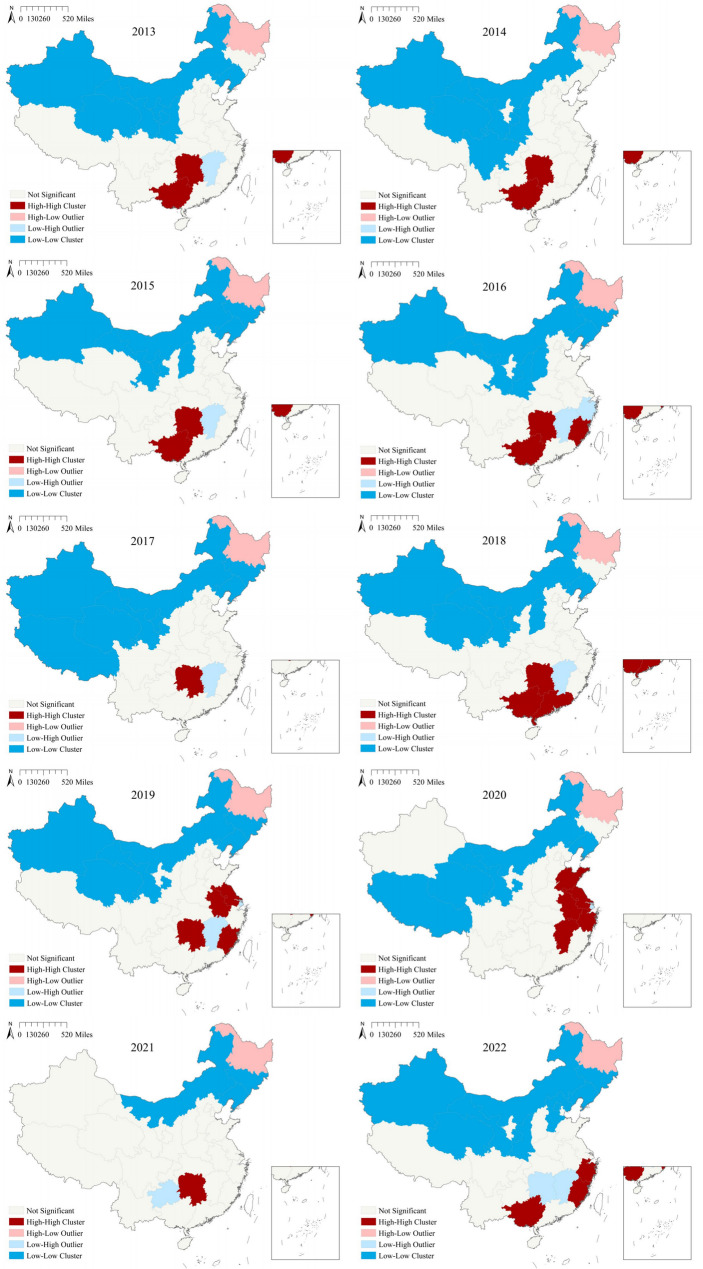
Local spatial autocorrelation analysis of ILI outbreaks in Chinese mainland, 2013–2022

The maps of LISA (Fig. 4) showed that during 2013–2015, high-high clusters were observed in the southern region, encompassing Guangxi and Hunan. From 2016 to 2019, Fujian, Guangdong, Jiangsu and Anhui successively joined the high-high clusters. In 2020, a high-high cluster was detected in the eastern part of the mainland, comprising Shandong, Jiangsu, Anhui, Zhejiang and Jiangxi. In 2021, the high-high cluster was confined solely to Hunan. By 2022, it encompassed Guangxi, Fujian and Zhejiang. Over the period of 2013 to 2022, low-low clusters predominantly occurred in the northern regions of the mainland, including Xinjiang, Inner Mongolia, Liaoning, Qinghai, Gansu, Ningxia, Shaanxi, Jilin, Shanxi, Tibet and other regions.

### Space-time scanning analysis

**Fig. 5:**
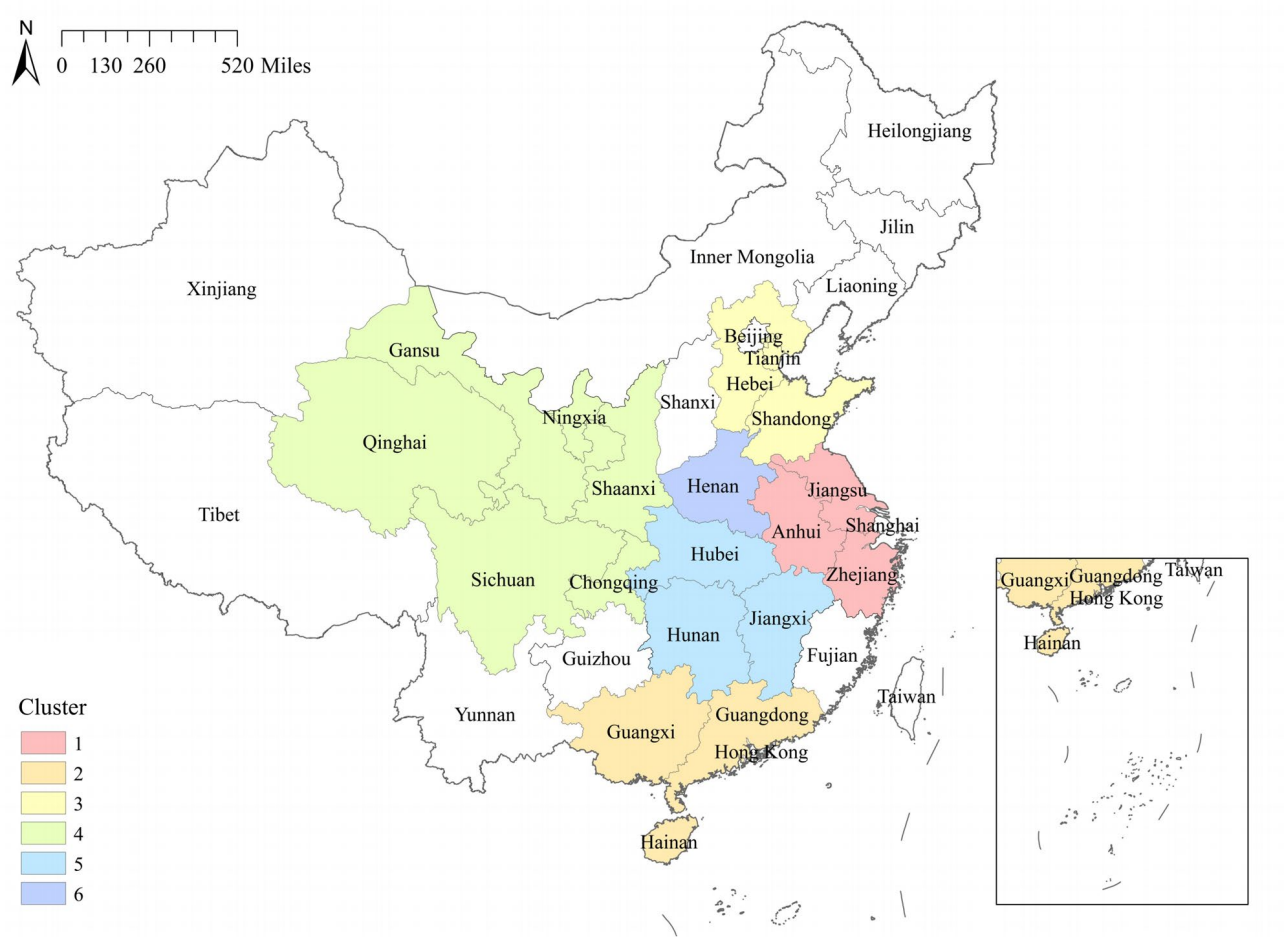
Spatio-temporal high-risk clusters of ILI outbreaks in Chinese mainland, 2013–2022

The spatio-temporal high-risk clusters for ILI outbreaks in Chinese mainland identified by the spatio-temporal scanning method were shown in Table 2; Fig. 5. Six high-risk clusters were detected from 2013 to 2022. The first cluster encompassed Jiangsu, Anhui and Zhejiang from December 2, 2019 to January 6, 2020 (RR = 42.41, *P* < 0.001). The second cluster was from November 20, 2017 to January 6, 2020, covering Guangdong, Hainan and Guangxi (RR = 5.72, *P* < 0.001). Shandong, Hebei and Tianjin were identified as the third cluster from November 27 to December 25, 2017 (RR = 21.42, *P* < 0.001). The fourth cluster involved Gansu, Qinghai, Ningxia, Shaanxi, Sichuan and Chongqing from November 11 to December 30, 2019 (RR = 13.32, *P* < 0.001). The fifth cluster was from November 25 to December 30 of 2019, including Jiangxi, Hubei and Hunan (RR = 16.03, *P* < 0.001). The sixth cluster was in Henan from December 9 to December 23, 2019 (RR = 9.16, *P* < 0.001).

**Table 2:** Retrospective spatio-temporal scan analysis of the cases of ILI outbreaks in Chinese mainland, 2013–2022

Cluster	Dates	Main areas in cluster	Coordinates, radius (km)	RR	LLR	*P*
1	2019/12/2–2020/1/6	Jiangsu, Anhui, Zhejiang	(32.04 N, 118.77E), 235.30	42.41	2316.56	< 0.001
2	2017/11/20–2020/1/6	Guangdong, Hainan, Guangxi	(23.13 N, 113.28E), 508.60	5.72	1752.69	< 0.001
3	2017/11/27–2017/12/25	Shandong, Hebei, Tianjin	(36.68 N, 117.00E), 272.74	21.42	674.28	< 0.001
4	2019/11/11–2019/12/30	Gansu, Qinghai, Ningxia, Shaanxi, Sichuan, Chongqing	(36.06 N, 103.82E), 767.02	13.32	582.14	< 0.001
5	2019/11/25–2019/12/30	Jiangxi, Hubei, Hunan	(28.68 N, 115.89E), 289.35	16.03	495.54	< 0.001
6	2019/12/9–2019/12/23	Henan	(34.76 N, 113.67E), 0	9.16	50.25	< 0.001

### Spatio-temporal evolution

**Fig. 6:**
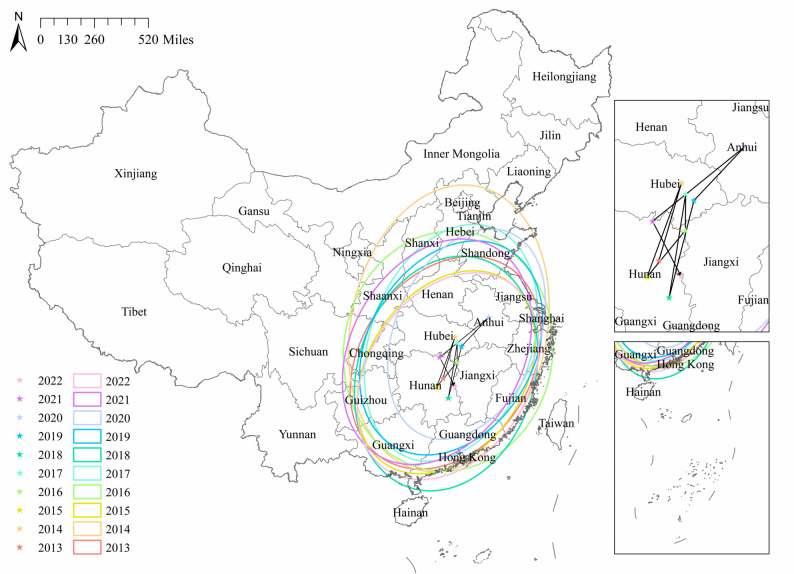
The results of median center and standard deviational ellipse of ILI outbreaks in Chinese mainland, 2013–2022

The results of the median center and standard deviational ellipse were presented in Fig. 6. During the study period, the median center of ILI outbreaks in Chinese mainland primarily resided in the central region. In 2013, it was located in Hunan, after which it migrated among Hubei, Hunan, Jiangxi, and Anhui in the subsequent years, eventually returning to Hunan in 2022. In the evolution of the ellipse representing the distribution of ILI outbreaks, the principal regions consistently lay in the eastern and central parts of Chinese mainland, encompassing the vast majority of areas in Guangdong, Guangxi, Guizhou, Hunan, Jiangxi, Fujian, Chongqing, Hubei, Anhui, Zhejiang, Jiangsu, Henan, Hebei, Shandong, Shaanxi, Shanxi and other regions. The ellipse initially exhibited a southwest-northeast extension, but over time, its major axis shortened, and the surrounding area decreased, indicating that the distribution of ILI outbreaks was gradually concentrated and dense.

### Exploration of influential factors

The results of multicollinearity testing using VIFs for all covariates were presented in Table 3. Among them, the VIF values of urbanization rate and disposable income per capita exceeded 10, therefore, these two variables were excluded. The remaining variables were re-tested for multicollinearity, and the results showed that all VIF values were below 10, allowing their inclusion in the final model for analysis. Models III containing spatial terms, a temporal term and a spatio-temporal interaction term were selected as the preferred models due to the lowest DIC (Table S1). The results of the analysis of factors influencing ILI outbreaks were shown in Tables 4 and 5.

**Table 3:** Multicollinearity evaluation results

Covariates	VIF (before)	VIF (after)
Temperature (℃)	4.84	4.73
Surface pressure (hPa)	4.19	4.07
Sunshine duration (h)	3.97	3.87
Precipitation (mm)	1.81	1.81
Relative humidity (%)	3.52	3.30
Wind speed (m/s)	1.48	1.40
Population density (1/km^2^)	4.58	3.95
Male (%)	1.64	1.55
Age ≤ 14 year (%)	3.43	1.88
Aging rate (%)	3.04	2.77
Urbanization rate (%)	12.34	Exclude
GDP per capita (yuan)	9.30	2.96
Disposable income per capita (yuan)	16.70	Exclude
Number of health technicians per 1000 people	3.82	2.60
Floating population (10,000 people)	2.60	2.50

**Table 4:** Univariate analysis of socioeconomic and climate factors associated with ILI outbreaks in Chinese mainland, 2013–2022

Covariates	Mean	SD	RR	95% CI
Temperature (℃)	−0.065	0.007	**0.937**	**0.925–0.949**
Surface pressure (hPa)	0.015	0.003	**1.015**	**1.010–1.022**
Sunlight duration (h)	−0.197	0.023	**0.821**	**0.786–0.859**
Precipitation (mm)	0.039	0.006	**1.040**	**1.026–1.052**
Relative humidity (%)	0.011	0.002	**1.011**	**1.007–1.016**
Wind speed (m/s)	−0.236	0.044	**0.790**	**0.725–0.860**
Population density (1/km^2^)	0.558	0.156	**1.744**	**1.290–2.387**
Male (%)	0.004	0.008	1.004	0.989–1.019
Age ≤ 14 year (%)	0.004	0.027	1.004	0.953–1.059
Aging rate (%)	−0.035	0.025	0.966	0.919–1.013
GDP per capita (yuan)	0.895	0.217	**2.447**	**1.600–3.743**
Number of health technicians per 1000 people	2.115	0.454	**8.290**	**3.404–20.206**
Floating population (10,000 people)	0.771	0.105	**2.162**	**1.758–2.656**

**Table 5:** Multivariate analysis of socioeconomic and climate factors associated with ILI outbreaks in Chinese mainland, 2013–2022

Covariates	Mean	SD	RR	95% CI
Temperature (℃)	−0.043	0.007	**0.958**	**0.945–0.972**
Surface pressure (hPa)	0.005	0.003	**1.005**	**1.000–1.011**
Sunshine duration (h)	−0.138	0.043	**0.871**	**0.801–0.947**
Precipitation (mm)	0.014	0.009	1.014	0.997–1.030
Relative humidity (%)	−0.009	0.003	**0.991**	**0.984–0.997**
Wind speed (m/s)	−0.198	0.046	**0.820**	**0.748–0.899**
Population density (1/km^2^)	0.254	0.199	1.292	0.866–1.895
Male (%)	0.022	0.008	**1.022**	**1.006–1.039**
Age ≤ 14 year (%)	0.110	0.028	**1.116**	**1.054–1.179**
Aging rate (%)	−0.032	0.025	0.969	0.923–1.017
GDP per capita (yuan)	0.653	0.235	**1.923**	**1.212–3.047**
Number of health technicians per 1000 people	0.527	0.511	1.692	0.623–4.623
Floating population (10,000 people)	0.664	0.129	**1.943**	**1.507–2.499**

In the univariate analysis, temperature, sunshine duration, and wind speed were negatively associated with ILI outbreaks. For every 1℃ increase in temperature, the risk of ILI outbreaks would decrease by 6.3% (95% CI: 0.925–0.949). An additional hour of sunshine was associated with a 17.9% (95% CI: 0.786–0.859) reduction in the risk of ILI outbreaks. Moreover, each increment of 1 m/s in wind speed led to an 21% (95% CI: 0.725–0.860) decrease in the risk of ILI outbreaks. Conversely, surface pressure, precipitation, relative humidity, population density, GDP per capita, the number of health technicians per thousand people and floating population were positively correlated with ILI outbreaks. A rise of 1 hPa in surface pressure was accompanied by a 1.5% (95% CI: 1.010–1.022) increase in the risk of ILI outbreaks. An additional 1 mm of precipitation was associated with a 4% (95% CI: 1.026–1.052) increase in the risk, while each percentage point increase in relative humidity corresponded to a 1.1% (95% CI: 1.007–1.016) increase in risk. Furthermore, regions with higher population density (RR = 1.744, 95% CI: 1.290–2.387), GDP per capita (RR = 2.447, 95% CI: 1.600–3.743), more health technicians per thousand people (RR = 8.290, 95% CI: 3.404–20.206) and larger floating population (RR = 2.162, 95% CI: 1.758–2.656) exhibited higher risks of ILI outbreaks.

In the multivariate analysis, temperature (RR = 0.958, 95% CI: 0.945–0.972), sunshine duration (RR = 0.871, 95% CI: 0.801–0.947), and wind speed (RR = 0.820, 95% CI: 0.748–0.899) remained protective factors against ILI outbreaks. Additionally, relative humidity (RR = 0.991, 95% CI: 0.984–0.997) also showed negative associations with ILI outbreaks. On the other hand, surface pressure (RR = 1.005, 95% CI: 1.000–1.011), male proportion (RR = 1.022, 95% CI: 1.006–1.039), the proportion of population aged 14 and below (RR = 1.116, 95% CI: 1.054–1.179), GDP per capita (RR = 1.923, 95% CI: 1.212–3.047), and floating population (RR = 1.943, 95% CI: 1.507–2.499) emerged as risk factors for ILI outbreaks.

**Fig. 7:**
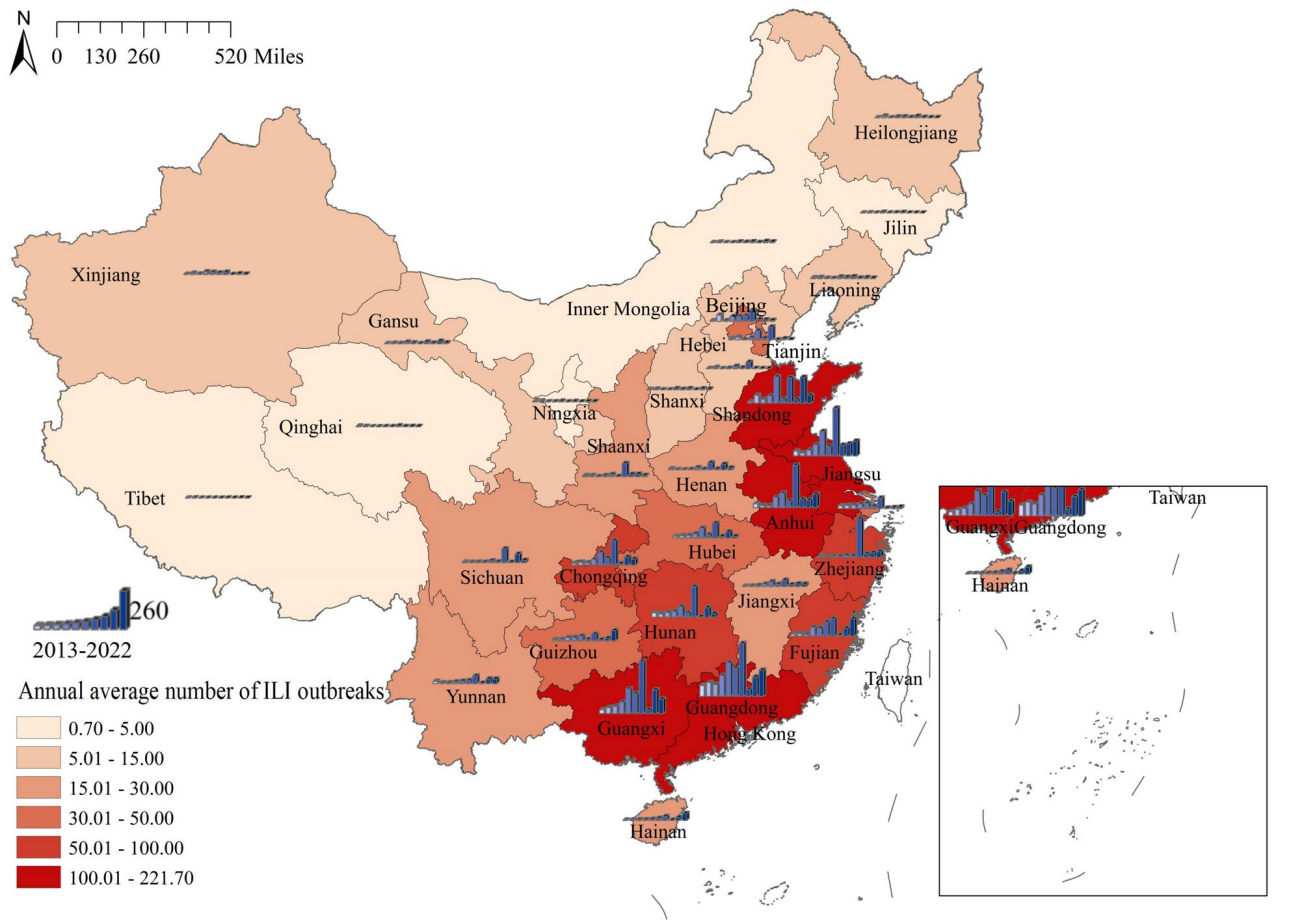
The spatial distribution of posterior means of risk ratio of ILI outbreaks based on Bayesian spatio-temporal models in Chinese mainland, 2013–2022

The spatial distribution of the posterior mean relative risks for ILI outbreaks was depicted in Fig. 7 and Table S2. The results showed that Guangdong, Guangxi, Jiangsu, and Anhui exhibited higher posterior mean relative risks, followed by Shandong, Chongqing, Hunan, and Fujian. And this distribution was similar to that of the spatial distribution of ILI outbreaks.

## Discussion

To the best of our knowledge, this study is the first to systematically demonstrate the long-term trend, seasonality, and spatio-temporal clustering distribution of ILI outbreaks in Chinese mainland, and to explore the influence of socioeconomic and meteorological factors on them.

According to the time series statistics of ILI% and positive detection rate in northern and southern regions of China, the study found that the incidence of influenza was cyclical and seasonal, with high incidence in winter and spring in the north, and concentrated in winter and summer in the south, which was similar to the previous findings [13]. The proportion of influenza subtypes also displayed marked seasonality in both southern and northern regions, a clear epidemiological pattern that has been reported previously [14]. Experimental studies demonstrated that the aerosol transmission of influenza viruses was dependent upon the relative humidity and temperature of the environment, with colder and drier conditions favoring viral spread [22, 23].

Based on the statistical analysis of the number of weekly ILI outbreaks in each province, municipality, and autonomous region in Chinese Mainland from 2013 to 2022, the study demonstrated a clear seasonality in ILI outbreaks. In northern regions, ILI outbreaks were fewer in number and predominantly occurred during winter. By contrast, southern areas experienced a greater number of ILI outbreaks, primarily concentrated in the winter and spring. Among them, Guangdong, Guangxi, Shandong, Jiangsu, and Anhui exhibited significantly higher numbers of outbreaks compared to other areas. From 2013 to 2019, the count of ILI outbreaks generally showed an oscillatory upward trend. This trend may be partly attributed to the increase in the number of surveillance sites. Meanwhile, population growth and mobility, economic development, and improvements in healthcare capacity collectively contributed to the enhanced case detection rates. These factors may have led to the observed upward trend in reported cases during this period. After peaking in 2019, the count of ILI outbreaks exhibited a downward trend. This decline can likely be attributed to the outbreak of COVID-19, following which the Chinese government implemented stringent non-pharmaceutical interventions. These measures were widely embraced by local authorities and the public, who diligently adopted protective behaviors, minimizing unnecessary outings, leading to a decrease in ILI outbreaks beginning in 2020 [24, 25]. Analogously, in other countries and regions, a substantial reduction in influenza activity was observed during the COVID-19 pandemic [26, 27]. However, with the relaxation of non-pharmaceutical interventions in China, the number of ILI outbreaks rebounded in 2021 and 2022 [25]. Consequently, the overall situation regarding influenza in China remains grave. Influenza vaccination is considered the most efficacious preventive measure. Currently, the widely used influenza vaccines in the domestic market are trivalent formulations recommended by the World Health Organization (WHO), containing antigenic components against A/H3N2, A/H1N1, and B/Victoria [28]. Although the vaccine components align with circulating viral strains, the fact that influenza vaccination in China is an out-of-pocket expense contributes to low vaccination rates and inadequate coverage, which in turn exacerbates the annual increase in influenza cases.

The study found significant regional disparities in ILI outbreaks across China, with spatial and temporal heterogeneity evident among different regions. Local spatial autocorrelation analysis indicated that high-high clusters were predominantly concentrated in the east and central-south regions of China, encompassing Guangdong, Guangxi, Hunan, Jiangsu, Anhui, Zhejiang, Fujian, and other economically developed and densely populated areas. Spatio-temporal scanning for high-risk zones during 2017–2020 identified similar regions, with a focus on Jiangsu, Anhui, Zhejiang, Guangdong, Guangxi and others. Median center and standard deviational ellipse analyses revealed that, throughout the spatio-temporal evolution of ILI outbreaks, the main regions consistently lay in the eastern and central parts of the Chinese mainland, consistent with former findings. Research by Zhang et al. demonstrated that influenza high-risk areas were concentrated in central China [12], while Shao et al.'s work showed that southeastern coastal regions were high-incidence areas and the most critical hot-spots for influenza [29]. Deng et al.'s study revealed that the primary aggregation zones for influenza were distributed across eastern and central China [30]. These findings provided corroboration for our study.

This study investigated the impact of meteorological factors on the risk of ILI outbreaks. The results showed a negative correlation between temperature and ILI outbreaks, with lower temperature corresponding to a higher risk of ILI outbreaks, consistent with the influence of temperature on influenza morbidity [14, 31]. Experimental studies had indicated that influenza viruses were more stable at lower temperatures, potentially due to an increased half-life of the viruses under cold conditions, reduced protease activity, decreased mucus production and cilia movement, suppression of defenses and immune responses against infection, ultimately affecting host susceptibility and facilitating increased viral transmission during winter [32, 33]. Furthermore, this study also revealed that sunshine duration and wind speed were protective factors against ILI outbreaks. Regarding sunshine duration, on the one hand, solar ultraviolet radiation could inactivate viruses in the environment [34]. On the other hand, sunshine exposure influenced immune function by altering the production of endogenous specific immunomodulatory agents [35], direct skin exposure to ultraviolet radiation might facilitate the generation of vitamin D3, which served as a regulator of the human immune system [36, 37]. With regard to wind speed, research findings were inconsistent [33, 38, 39]. Some studies suggested that higher wind speeds prolonged the duration and broadened the geographical reach of viral transmission [33, 38], whereas others reported that calm or low wind speeds significantly increased the risk of influenza activity [39, 40], aligning with our findings. One study found that higher wind speeds significantly reduced the number of infectious airborne particles in the environment, thereby decreasing the likelihood of exposure to infective particles and lowering the risk of infection [41]. Surface pressure emerged as a risk factor for ILI outbreaks, consistent with findings from other studies [40, 42]. This may be attributed to the fact that high pressure often accompanies dry weather, promoting indoor activities and increased interpersonal interactions, potentially heightening the risk of influenza transmission. Additionally, precipitation was positively associated with ILI outbreaks, although research conclusions on this matter were inconsistent [11, 43, 44]. One study indicated that heavier rainfall could lead to increased indoor crowding, raising the risk of contact-based transmission [45]. Relative humidity was also related to ILI outbreaks, showing a positive association in univariate analysis but a negative association in multivariate analysis, possibly influenced by other factors. Various studies demonstrated that low indoor and outdoor humidity facilitated enhanced viral survival in respiratory droplets, thus promoting influenza virus spread [22, 46]. The effect of relative humidity on influenza virus stability may lie in changes in salt concentration within droplets: at high relative humidity (99%−100%, or physiological conditions), where physiological salt concentrations remain constant, viruses tend to remain stable; at lower relative humidity (50%−99%), salt concentration increases due to evaporation of the droplets may cause damage to influenza viruses; and at the lowest relative humidity (< 50%), the salt crystallizes, maintaining the stability of the viruses [47].

The study also explored the influence of socioeconomic factors on the risk of ILI outbreaks. The results showed that population density, GDP per capita, the number of health technicians per thousand people and floating population were positively correlated with the ILI outbreaks, which was consistent with findings from other studies [11, 48, 49]. These suggest that in regions with better economic conditions, there tends to be a larger population, higher levels of accessible healthcare, and a greater propensity for residents to seek medical attention proactively, which may lead to higher influenza diagnosis rates and consequently elevate the risk of ILI outbreaks. Furthermore, multivariate analysis revealed a positive association between the male proportion and the proportion of population aged 14 and below with ILI outbreaks. The research has indicated that compared to females, males have weaker immune systems, particularly when it comes to common viral respiratory infections, making them more susceptible to infection, experiencing more severe symptoms and longer duration, and being more likely to require hospitalization due to influenza [50]. Moreover, the infection rate and prevalence of the influenza virus were highest among children [51], aligning with our findings.

The study investigated the spatio-temporal patterns of ILI outbreaks across different time and space from 2013 to 2022. A Bayesian model incorporating spatial, temporal, and spatio-temporal interaction terms was constructed to closely examine the heterogeneity of ILI outbreaks at the provincial level, with the aim of obtaining robust and comparable posterior estimates. By adjusting for various meteorological and socioeconomic variables and incorporating spatio-temporal effects, the validity of our findings has been strengthened.

This study also acknowledges several limitations that warrant consideration when interpreting its findings. Firstly, the sample size, while sufficient for initial exploration, was relatively modest and geographically concentrated, potentially limiting the generalizability of the results across broader or more diverse populations. Secondly, the data used in this study are surveillance data, verified and reported by county or district level disease prevention and control institutions through the “China Influenza Surveillance Information System”, so the possibility of under-reporting cannot be ruled out. Thirdly, the cross-sectional design employed here establishes associations but cannot determine causality or elucidate the temporal dynamics between the variables studied. Finally, while key variables were controlled for, the potential influence of unmeasured confounding factors cannot be entirely ruled out. Therefore, future research would benefit from longitudinal designs, larger and more heterogeneous samples, incorporating objective measurements where feasible, and exploring a wider range of potential covariates to strengthen causal inference and enhance external validity.

## Conclusion

The study revealed distinct patterns and related influencing factors of ILI outbreaks in Chinese mainland from 2013 to 2022. Seasonality emerged as a key characteristic, with northern regions experiencing predominant winter outbreaks, while southern regions faced more frequent outbreaks spanning winter and spring, reflecting potential climatic and environmental influences on viral transmission dynamics. Spatially, high clustering of ILI outbreaks was concentrated in populous and economically developed provinces, including Guangdong, Guangxi, Shandong, Jiangsu, Anhui, Zhejiang, and Fujian, highlighting the role of regional disparities in population density, connectivity, and resource distribution. This spatio-temporal patterns of ILI outbreaks could be explained by socioeconomic and meteorological factors to a certain extent. It is recommended to strengthen influenza surveillance, optimize resource allocation, and enhance vaccination efforts in the high-risk areas identified in this study, effectively preventing the exacerbation and spread of ILI outbreaks during peak seasons and in high-incidence regions.

## Supplementary Information

Supplementary Material 1.

Supplementary Material 2.

## Data Availability

The data are available through the corresponding author upon reasonable request.

## Abbreviations

CI: Confidence interval
COPD: Chronic obstructive pulmonary disease
COVID-19: Corona Virus Disease 2019
DIC: Deviance information criterion
GDP: Gross domestic product
ILI: influenza-like illness
LISA: Local indicators of spatial association
LLR: Log Likelihood Ratio
RR: relative risk
VIF: variance inflation factor
WHO: World Health Organization
ZINB: Zero-inflated Negative Binomial
