# Supplementary material for: Spatio-temporal patterns and associated factors of influenza-like illness outbreaks in Chinese mainland: a Bayesian modeling study
Source: BMC Public Health. 2025 Dec 18;26:283. doi: 10.1186/s12889-025-25615-7 (PMC12828928; doi:10.1186/s12889-025-25615-7)
Supplement: Supplementary file 1 — Supplementary Material 1. [file 12889_2025_25615_MOESM1_ESM.docx]

**Table S1.** Model performance and selection.

| **Model** | **Type** | **Included terms** | **DIC *** |
| --- | --- | --- | --- |
| Model I | Spatial model | $u_{i}+v_{i}$ | 26844.16 |
| Model II | Spatial + temporal model | $\text{u}_{\text{i}}\text{+}\text{v}_{\text{i}}\text{+}\text{φ}_{\text{t}}$ | 22572.48 |
| Model III | Spatial + temporal + spatio-temporal interaction model | $\text{u}_{\text{i}}\text{+}\text{v}_{\text{i}}\text{+}\text{φ}_{\text{t}}\text{+}\text{ψ}_{\text{it}}$ | 22355.93 |

* Deviation information criteria evaluating the goodness of fit of the models for ILI outbreaks.

**Table S2**. Variance decomposition analysis from Model III for ILI outbreaks in Chinese mainland, 2013–2022.

| **Component** | **Variance** | **Proportion (%)** |
| --- | --- | --- |
| Meteorological factors | 0.0041 | 0.21 |
| Socio-economic indicators | 0.3714 | 18.47 |
| Spatial term | 0.6953 | 34.59 |
| Temporal term | 0.5627 | 27.99 |
| Spatio-temporal interaction term | 0.3527 | 17.54 |
| Residual | 0.0242 | 1.20 |
